# Childhood acute poisoning in the Italian North-West area: a six-year retrospective study

**DOI:** 10.1186/s13052-020-00845-0

**Published:** 2020-06-11

**Authors:** Giovanni N. Berta, Federica Di Scipio, Francesca M. Bosetti, Barbara Mognetti, Federica Romano, Maria E. Carere, Anna C. Del Giudice, Emanuele Castagno, Claudia Bondone, Antonio F. Urbino

**Affiliations:** 1 Department of Clinical and Biological Sciences, Pharmacology Unit, University of Turin, Turin, Italy; 2 Division of Pediatrics, ASL AT, Cardinal Massaia Hospital, Asti, Italy; 3 Department of Life Science and Systems Biology, University of Turin, Turin, Italy; 4 Department of Surgical Sciences, C.I.R. Dental School, University of Turin, Turin, Italy; 5 Pediatric Unit 1, Maria Vittoria Hospital, Turin, Italy; 6 Department of Pediatric Emergency, Regina Margherita Children’s Hospital, A.O.U. Città della Salute e della Scienza di Torino, Turin, Italy

**Keywords:** Poisoning, Pediatric, Childhood, Toxicovigilance

## Abstract

**Background:**

Data about acute poisoning in Italian pediatric patients are obsolete or absent. This study would partially fill this exiting gap and compare the scene with others around the world.

**Methods:**

A retrospective evaluation was performed on a 2012–2017 data registry of the Children’s Emergency Department at the Regina Margherita Hospital of Turin, where 1030 children under age 14 were accepted with a diagnosis of acute intoxication.

**Results:**

The median age of the patients was 2.2 years (IQR 2.3) and 55% were male. Events occurred mostly in children aged 1–4 years (*n* = 751, 72.9%). Six hundred and eight patients (59%) were exposed to Nonpharmaceutical agents, the household cleaning products being the more frequent (*n* = 298, 49%). Exposure to Pharmaceuticals were 422 (41%); the most common Pharmaceuticals were analgesics (*n* = 88, 20.8%), psychotropics (*n* = 77, 18.2%) and cardiovascular (*n* = 53, 12.6%) drugs. The 85% of the intoxications occurred accidentally, the 10.6% as therapeutic error, the 2.3% as suicide attempts and the 1.5% for recreational purposes. No patient died.

**Conclusions:**

Despite acute poisoning being a relevant problem in pediatric emergency, our results would seem to paint a less worrying picture if compared to other countries, mainly when considering the children hospitalized in the pediatric intensive care unit and the number of deaths. Nevertheless, our study might represent a tool for public health authorities to program incisive interventions.

## Introduction

Acute exposure to chemicals is a common event in pediatrics: it represents one of the leading causes of morbidity and mortality around the world [1, 2]. Every year, the entry of over 2000 new chemical substances into the environment increases the risk of intoxication [3]. In 2017, the 35^th^ Annual Report of the American Association of Poison Control Centers’ National Poison Data System referred more than two million calls for cases of human exposure to toxic substances, of which more of 50% concerned acute intoxications in children under 13 years of age [4].

The evident curiosity for the surroundings and the desire to know, to explore and to emulate adults [5], make children particularly exposed to acute intoxications, while above 10 years intoxication is often intentional for suicide/demonstration purposes. In addiction, a considerable proportion of acute poisonings can be attributed to therapeutic errors or erroneous administrations by parents or caregivers [4, 6]. According to a retrospective analysis led in the US, every year nearly 64,000 errors in drug administration occur, the majority of which in their own homes [7].

However, it is difficult to estimate the real incidence of acute poisoning in children due to the relatively small number of studies available in literature: furthermore, to the best of our knowledge, no recent information on acute intoxication in childhood in Italy is available. The last relevant publication dates back to 1998 and is based on a series of data collected from 1975 to 1994 from the Northeast of the Nation [8].

For the above mentioned reasons, we performed this retrospective study to examine the demographic and the epidemiological data of children aged from 0 to 14 years entering the Children’s Emergency at the Regina Margherita Hospital (CE-RMH) of Turin with a diagnosis of acute intoxication during a period of 6 years (from January 1^st^, 2012 to December 31^st^, 2017), principally to determine the responsible agents and the circumstances that led to the exposure within the different age classes and to describe the type of treatment and the final outcome. The knowledge of these variables would allow to paint the actual situation in our area and to compare it with others around the world, and to identify/select specific preventive strategies.

## Materials and methods

### Design and setting

This is a retrospective study on ascertained acute poisonings on consecutive patients aged from 0 to 14 who presented at the Regina Margherita Hospital from January 1^st^, 2012 to December 31^st^, 2017. This is an inner-city teaching tertiary hospital in Turin, Piedmont, that serves local residents and out-of-state referrals with an estimated catchment area of about 500,000 potential users, being therefore one of the largest children’s hospitals in Italy.

### Ethical considerations

This study was approved by the Ethics Review Board of Hospital (Comitato Etico Interaziendale A.O.U. Città della Salute e della Scienza di Torino, number 00156/2019) in compliance with the Italian regulations in force for data collection and in accord with the principles of declaration of Helsinki.

### Data collection

The unidentified information and related data on individual patients were obtained from the database of Hospital discharge cards reporting. Being the study retrospective, data extractors were not aware of the hypothesis investigated. All data are available in Figshare Public repository. (Accession number 10.6084/m9.figshare.11382054.v1). Patients were not involved in the design, or conduct, or reporting, or dissemination plans of our research.

Patients were identified by Emergency Department physicians with the following data collected via interviews of patients and caregivers: personal and demographic information (age, gender, nationality), date and time of admission to the CE-RMH, temporary observation (if any), number (one or more) and type of substance involved, route of exposure (oral, ocular, cutaneous, inhalation, parenteral), previous episodes of poisoning, mode of poisoning (suicide attempt, intentional recreational, accidental, therapeutic error), packaging (non-original packaging/free tablets or original packaging/appropriate location), possible action of caretakers before arrival at the emergency room (cleansing, induction of vomiting, giving food of water or milk), request of advice from the National Poison Control Center (CAV) by the CE-RMH, type of treatment (medical or surgical), therapies, outcome (discharge or hospitalization), possible post-discharge home therapy. No follow-up was carried out. In some considered variables, the number of patients may be lower than 1030 due to missing values.

Substances were classified in two categories: Pharmaceuticals and Nonpharmaceuticals. Pharmaceuticals consist of analgesics (including anti-inflammatories, opioid analgesics and antispasmodics), psychotropics (including barbiturates, benzodiazepines, neuroleptics, antidepressant), respiratory drugs, antimicrobials (antibacterials, antivirals and antifungals), cardiovascular drugs (including diuretics), antihistamines, hormones and gastrointestinal drugs, vitamins, supplements and herbal remedies. The Nonpharmaceutical agents include alcohol, carbon monoxide (CO)/fumes, household cleaning products, plants/mushrooms, cosmetics, recreational drugs, pet food and viper bite. Adverse drug reactions, food intoxications and chronic poisoning were excluded from the study.

Four age groups were defined according to psychophysical development: i) under 1 year of age; ii) 1 to 4 years old; iii) 5 to 9 years old; iiii) 10 to 14 years old.

At arrival at the CE-RMH, patients were tagged according to the seriousness of their clinical situation and identified with a colored code according to the Italian guidelines for the correct execution of triage in emergency rooms: i) white, no urgency, no alteration of vital functions, no critical symptomatology or at risk of aggravation; ii) green, minor emergency, at the time of evaluation vital functions are intact and vital parameters in the norm; iii) yellow, serious emergency, patients with impending threat of failure of the vital functions (consciousness, breath, circulation); iiii) red, life threatening.

### Data analysis

Statistical analysis of the data was performed using the Statistical Package for the Social Sciences (SPSS), version 24.0 software (SPSS, Inc., Chicago, IL, USA). Values of quantitative variables were expressed as mean ± standard deviation or median and interquartile range (IQR), while values of categorical variables were presented as frequencies and percentages. The χ2 test was used to evaluate any potential association between categorical variables and the Mann-Whitney U-test or the Kruskal-Wallis test were used to assess differences of quantitative variables between two or more groups of children, as appropriate. When there were significant differences according to the Kruskal-Wallis test, pairwise multiple comparisons were carried out using the Dunn test. Multiple logistic regression models were developed to identify predictors of hospitalization (yes vs. no) and severity of clinical status (minor -white or green code-, vs. serious - yellow and red codes). Purposeful selection of statistically (*p* values ≤0.2 in the univariate analyses) and clinically relevant was conducted. Data were presented as odds ratio (OR) and 95% confidence intervals (CI). Two-tailed *p* values less than 0.05 were considered statistically significant.

## Results

### Patients

**Fig. 1:**
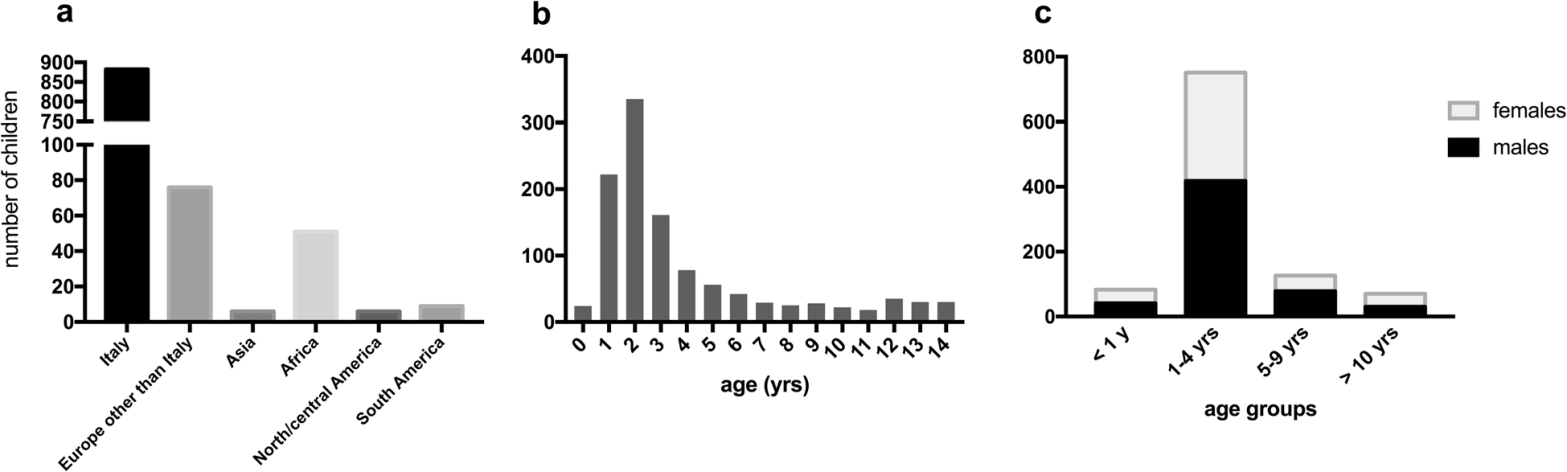
Epidemiological data. Distribution of the population is summarized according to geographical origin (**a**), age (**b**) and sex according to the four age categories described in Material and Methods (**c**)

From January 1^st^, 2012 to December 31^st^, 2017, a total of 1030 (567 males and 463 females) consecutive acute intoxicated patients below the age of 14 years (mean 3.4 ± 3.1, median 2.2 and IQR 2.3) presented to the Children’s Emergency at the Regina Margherita Hospital (CE-RMH). These represent the 0.38% of the total accesses at the CE-RMH in the same period (*n* = 271,654 patients). Their distribution according to the countries of origin and age are detailed in Fig. 1a and b, respectively. Children were grouped in the above-mentioned age-categories as reported in Fig. 1c. Under the age of 9, males and females equally accessed the CE-RMH; on the contrary, over the age of 10, the majority of cases were girls (57.1%, *p* = 0.05 males vs. females).

### Patient access to the Children’s emergency room

An average of 85.8 ± 9.95 patients per month accessed the CE-RMH due to acute intoxication. Accesses were less frequent between 1 a.m. and 9 a.m. (only 5.6% of the total) and very frequent between 7 p.m. and 11 p.m. (37.7% of the total).

Children were tagged according to the severity of their clinical situations and identified with a colored code. Their distribution by age, type of agent and output is detailed in Table 1.

**Table 1:** Distribution of age, type of agent and hospitalization according to ER access color code

Color code	Number (%)	Age [median, (IQR)]	Type of agent [n, (%)]		Hospitalization [n, (%)]
			Pharmaceuticals	Nonpharmaceuticals	
White	10 (1%)	2.0 (4.2)	4 (40)	6 (60)	1 (10)
Green	88 (8.5%)	2.7 (3.5)	31 (35.2)	57 (64.8)	7 (7.9)
Yellow	920 (89.3%)	2.2 (2.1)	380 (41.3)	540 (58.7)	76 (8.3)
Red	12 (1.2%)	4.3 (11.5)	7 (58.3)	5 (41.7)	12 (100)

Children requiring a medical intervention (*n* = 982, 95.3%) had a median age of 2.2 years (IQR 2.4) and patients requiring surgery (*n* = 48, 4.7%) a median age of 2.1 years (IQR 1.9).

### Agents causing intoxication

The number of exposures to the subclasses of Non- and Pharmaceutical agents were stratified according to the age groups or sex and detailed in Table 2**.** Six hundred and eight patients (59%) were exposed to Nonpharmaceutical compounds, 63% of which were males. Among household cleansers, 96 cases were due to exposure to bleach or bleach-containing products, 55 to surface cleaners, 37 to dish washing liquids and 18 to drain cleaners. The more frequent intoxicating product among cosmetics was nail polish remover (16 out of 54 cases). Tobacco was responsible for 25 out of 30 exposure to recreational drugs.

**Table 2:** Nonpharmaceuticals and Pharmaceuticals distribution according to the age and sex of intoxicated children

	Age [n, (%)]				Sex [n, (%)]		Total
	< 1 years	1–4 years	5–9 years	> 10 years	Males	Females	
**Nonpharmaceuticals**							
Household cleansers	19 (6.4)	244 (81.9)	22 (7.4)	13 (4.4)	169 (56.7)	129 (43.3)	298
Cosmetics	3 (5.6)	47 (87)	1 (1.8)	3 (5.6)	32 (59.2)	22 (40.8)	54
CO	6 (14.6)	10 (24.4)	19 (46.3)	6 (14.6)	17 (41.5)	24 (58.5)	41
Plants and mushrooms	3 (8.6)	26 (74.3)	4 (11.4)	2 (5.7)	17 (48.6)	18 (51.4)	35
Recreational drugs	7 (23.3)	21 (70)	0 (0)	2 (6.7)	20 (66.7)	10 (33.3)	30
Alcohol	0 (0)	7 (58.3)	2 (16.7)	3 (25)	7 (58.3)	5 (41.7)	12
Pet food	0 (0)	6 (66.7)	3 (33.3)	0 (0)	5 (55.5)	4 (44.5)	9
Viper bite	0 (0)	1 (25)	1 (25)	2 (50)	2 (50)	2 (50)	4
Other Nonpharmaceuticals	6 (4.8)	85 (68)	24 (19.2)	10 (8)	73 (58.4)	52 (41.6)	125
**Totals per age category or sex**	**44 (7.2)**	**447 (73.5)**	**76 (12.5)**	**41 (6.7)**	**342 (56.2)**	**266 (43.7)**	**608 (100)**
**Pharmaceuticals**							
Analgesics	11 (12.5)	62 (70.4)	9 (10.2)	6 (6.8)	51 (57.9)	37 (42.1)	88
Psychotropics	3 (3.9)	41 (53.2)	21 (27.3)	12 (15.6)	45 (58.4)	32 (41.6)	77
Cardiovascular drugs	3 (5.7)	42 (79.2)	5 (9.4)	3 (5.7)	27 (51)	26 (49)	53
Antimicrobials	5 (17.9)	23 (82.1)	0 (0)	0 (0)	10 (35.7)	18 (64.3)	28
Hormonal drugs	1 (3.8)	22 (84.6)	2 (7.7)	1 (3.8)	13 (50)	13 (50)	26
Respiratory drugs	1 (5.6)	14 (77.8)	2 (11.1)	1 (5.6)	7 (38.9)	11 (61.1)	18
Antihistamines	0 (0)	14 (87.5)	1 (6.2)	1 (6.2)	6 (37.5)	10 (62.5)	16
Gastrointestinal drugs	2 (18.2)	7 (63.6)	1 (9.1)	1 (9.1)	6 (54.5)	5 (45.5)	11
Vitamins/supplements	12 (36.4)	17 (51.5)	3 (9.1)	1 (3.0)	22 (66.7)	11 (33.3)	33
Other Pharmaceuticals	3 (4.2)	57 (79.2)	8 (11.1)	4 (5.6)	38 (52.8)	34 (47.2)	72
**Totals per age category or sex**	**41 (9.7)**	**299 (70.9)**	**52 (12.3)**	**30 (7.1)**	**225 (53.3)**	**197 (46.7)**	**422 (100)**

Fifty percent of alcohol and 37% of plant and mushroom intoxications occurred in summer, 63.3% of drug and tobacco intoxication in spring / summer.

Among analgesics, 48 children were exposed to acetaminophen, 21 to a FANS and 2 to opioid analgesics. Benzodiazepines were the most frequent agent among psychotropics (24 out of 77), 17 children were intoxicated by antiepileptics and 15 by antidepressants. Levothyroxine was responsible for 13 out of 26 exposures to hormonal drugs. Trend by day of the week, hours or season were not evidenced for Pharmaceutical intoxications.

In any age group, intoxications with Nonpharmaceuticals were more frequent than with Pharmaceuticals. In general, over the age of 10, intoxication in girls was more frequent than in males although at the limit of significance.

The number of poisoning substances and the percentage of hospitalizations are detailed in Table 3.

**Table 3:** Number of poisoning substances and hospitalization

Number of intoxicating substances	Number of patients (%)	Age [years, median (IQR)]	Hospitalization n, (%)
1	981 (95)	2.1 (2.3)	86 (8.8)
2	37 (3.6)	2.4 (6.5)	5 (13.5)
3 or more	12 (1.2)	3.4 (3.8)	5 (41.7)

When comparing children exposed to one single substance to the rest of the population considered, it appears that they were younger (median age 2.1 years, IQR 2.3) than children poisoned by two or more agents (median age 3.5 years, IQR 3.9, *p =* 0.005). Twenty-five patients (2.4%, 12 males and 13 females) had already experienced previous episodes of intoxication; their median age (3.9 years, IQR 11.6) was significantly higher than that of children intoxicated for the first time (2.2 years, IQR 2.3, *p <* 0.01) and ten of them were at least 10 years old with the higher probability to access as a red code (*p <* 0.001).

### Mode of exposure

**Fig. 2:**
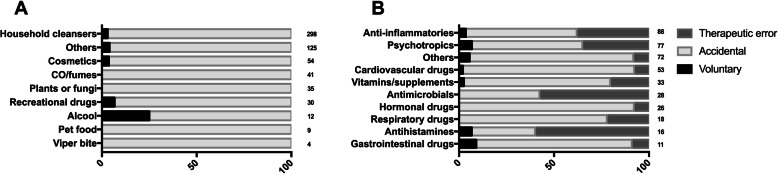
Mode of poisoning with principal classes of Nonpharmaceutical (**a**) and Pharmaceutical agents (**b**). Data are expressed as percentage of total cases per class (indicated at the end of each row). Differences between groups were always significant (*p* < 0.001) except accidental vs. therapeutic error (*p* = 0.055)

The mode of exposure to Nonpharmaceuticals and Pharmaceuticals is reported in Table 4, while the different classes are detailed in Fig. 2.

**Table 4:** Mode of exposure to Pharmaceuticals and Nonpharmaceuticals

	Mode of exposure [n, (%)]				Total [n, (%)]
	Accidentally exposed	Therapeutic error	Suicide attempt	Recreational purpose	
Nonpharmaceuticals	586 (96.4)	0	8 (1.3)	14 (2.3)	**608 (59)**
Pharmaceuticals	296 (70.1)	109 (25.8)	16 (4)	1 (0.2)	**422(41)**
**Total [n, (%)]**	**882 (85.6)**	**109 (10.6)**	**24 (2.3)**	**15 (1.5)**	**1030 (100)**

Detergents were the main cause of intoxication (Fig. 2a). Among the Pharmaceuticals (Fig. 2b), analgesics and psychotropics were the main cause of both accidental poisonings and therapeutic errors. Nevertheless, there was no significant correlation between any class of Pharmaceutical and the mode of exposure.

Twelve out of 16 suicide attempts were performed with Pharmaceutical agents by children older than 10, and 75% of them used psychotropics drugs.

Therapeutic errors were more frequent in children younger than 1 year (26/83 children younger than 1 year, 31.3%), while the 1–4 years old were mostly intoxicated accidentally (687/751 children aged 1–4, 91.5%). Notably, the only cases of intentional intoxication were found within the > 10 years old group. Children who attempted suicide were older (median 13.2 years, IQR 0.63) than those who had accidental exposure (median 2.1 years, IQR 1.9, *p <* 0.001), therapeutic errors (median 2.3 years, IQR 3.9, *p <* 0.001) and recreational purposes (median 12.6 years, IQR 10.7, *p <* 0.001); moreover, children who had recreational purposes were older than those who underwent accidental poisonings (*p <* 0.001) and therapeutic errors (*p <* 0.001). The ingestion resulted the most frequent route in all age groups (*p <* 0.001).

Although data were available only for 330 out of the 882 accidentally exposed patients, in 206 intoxications (62.4%) the product packaging was original, while in 51 situations (15.4%) it was not. Moreover, 41 children (12.4%) subtracted the agent from the appropriate place, while 32 (9.7%) unintentionally assumed the toxic agent considering it as a game. The main place of exposure resulted own home (*n* = 190, 18.4%), for 29 children (2.8%) it happened in public places, for 10 (1%) in a youth center (mainly with detergents and cosmetics) and for 5 (0.5%) at school (mostly with plants and ink). The route of exposure was oral in the majority of cases (*n* = 915, 88.8%), whereas inhalation and cutaneous contacts occurred in 49 (4.8%) and 20 (1.9%) children, respectively.

### Care and disposition of patients

Interventions of caretakers, if any, and dispositions are detailed in Table 5.

**Table 5:** Interventions of caretakers, dispositions and outcomes according to the exposure to Non- or to Pharmaceuticals.

	Nonpharmaceuticals [n, (%)]	Pharmaceuticals [n, (%)]
Caretakers reaction (*n*=161)		
*No reactions*	418 (68.4)	366 (86.7)
*Tried to remove the substance by cleaning*	55 (9)	12 (2.8)
*Gave water to drink*	31 (5.1)	7 (1.7)
*Gave milk to drink*	23 (3.8)	3 (0.7)
*Gave food*	0	3 (0.7)
*Induced vomiting*	13 (2.1)	14 (3.3)
Poison center consultation	511 (83.6)	349 (82.7)
Medical treatments	559 (91.9)*	416 (98.6)^#^
Surgeries	49 (8.1)*	6 (1.4)^#^
Home therapy	359 (59)^§^	95 (15.6)^§^
Hospitalization	58 (9.5)	40 (9.5)
Intensive care unit hospitalization	0	0
Other (leaving before consulting or discharge from hospital against medical advice)	7 (1.1)	1 (0.2)

* and # *p* < 0.001; § *p* = 0.001

In 33 out of the 608 cases of exposure to Nonpharmaceuticals, the intervention of caretakers is unknown, while the substance category in which the action was more frequent was detergents (101/161, 62.7%); no action was taken in the cases of carbon monoxide poisoning or in viper bite.

Fifty-one children, 17 of which poisoned by plants or mushrooms and 14 poisoned by recreational drugs, required activated charcoal therapy. Sixteen patients underwent gastric lavage, among which 4 exposed to detergents and 5 to recreational drugs. In one case of intoxication with a cleanser (sodium hypochlorite) it was necessary to administer an antidote (sodium thiosulfate).

The parents’ action was about two and a half times more frequent in the case of Nonpharmaceutical than in Pharmaceutical poisoning (*p <* 0.001). Among the situations requesting CAV advice, 69 events were due to analgesics, 58 to psychotropics and 44 to cardiovascular drugs. One hundred twenty-six children required activated carbon therapy, most frequently in case of contact with analgesics (*n* = 27) and cardiovascular drugs (*n* = 25). Moreover, 35 children, of whom 12 exposed to painkillers, underwent gastric lavage. In one intoxication by a benzodiazepine, an antidote (Flumazenil) was administered. In general, activated carbon therapy was used much more often in case of exposure to Pharmaceuticals than to Nonpharmaceuticals (29.8% vs 8.34%, *p =* 0.001). The same for gastric lavage (3.5 times more).

### Outcomes

Among exposure to Nonpharmaceuticals, detergents and household products represented the main cause of hospitalization (31 cases), while the Pharmaceuticals causing the highest number of hospitalizations were analgesics and psychotropics (13 and 10, respectively).

In general, discharge was more frequent than hospitalization (90.4% vs 9.6%) even if in patients over 10 years the percentage of hospitalization was significantly higher (41.2%) than in any other age group (*p <* 0.001). Among the 96 hospitalized patients, 78 intoxications occurred through the oral route, 11 by inhalation, 4 by dermal contact and 3 through parenteral/transdermal route (*p <* 0.05, oral vs. any other route). The largest number of hospitalizations occurred in the category of accidental exposure (57.6%) and 60% were due to Nonpharmaceuticals. In none of the 1030 cases of our series there was death.

### Predictors

The results of multivariable regression models for severity of clinical status, hospitalization and time spent at the ER are presented in Table 6. The odds of having a severe clinical status (yellow or red code) was higher in children requiring an advice from the CAV (OR 4.89, *p* < 0.001), and in those exposed to CO/fumes compared to Pharmaceutical poisoned children (OR 10.18, *p* = 0.029), while it was lower in those exposed to cosmetics (OR 0.40, *p* = 0.05). The hospitalization was three times more likely to occur in children from extra-European countries (*p* < 0.001) and in those intoxicated due to therapeutic error (OR 2.74, *p* = 0.01) compared to accidentally exposed children. The odds increased to 3.22 (*p* = 0.009) for cases of intentional poisoning.

**Table 6:** Multivariable regression for predictors of severity of clinical status and hospitalization. CI Confidence interval; OR Odds ratio; CO Carbon monoxide; CAV National Poison Control Center. ^a^Including plants/mushrooms, cosmetics, pet food and viper bite

	Seriousness of clinical status (serious vs. no serious)	Hospitalization (yes vs no)	Lenght of stay at the emergency room (at least 12 h vs less than 12 h)			
Predictor variables	Adjusted OR (95% CI)	*p* value	Adjusted OR (95% CI)	*p* value	Adjusted OR (95% CI)	*p* value
**Gender**						
Female	1		1		1	
Male	0.70 (0.45–1.10)	0.124	0.75 (0.57–0.98)	0.040	0.75 (0.56–0.99)	0.045
**Age in years**						
< 1	1.28 (0.40–4.09)	0.671	0.59 (0.59–1.37)	0.158	0.55 (0.24–1.28)	0.168
1–4	1.64 (0.62–4.31)	0.319	0.42 (0.18–0.98)	0.046	0.84 (0.42–1.66)	0.611
5–9	0.99 (0.337–2.91)	0.985	0.43 (0.16–1.15)	0.093	0.96 (0.45–2.05)	0.924
10–14	1		1		1	
**Nationality**						
European	–		1		–	
Extra-European	–		3.47 (1.82–6.62)	< 0.001	–	0.126
**Advice from CAV**						
No	1		1		1	
Yes	4.89 (3.01–7.93)	< 0.001	1.12 (0.58–2.16)	0.743	1.63 (1.06–2.50)	0.027
**Mode of poisoning**						
Accidental	1		1		1	
Intentional	2.48 (0.43–14.23)	0.309	3.22 (1.34–7.74)	0.009	3.3 (1.35–8.09)	0.009
Therapeutic error	0.595 (0.28–1.27)	0.178	2.74 (1.27–5.91)	0.010	1.91 (1.17–3.12)	0.008
**Number of substances**						
1	1		1		1	
≥ 2	5.56 (1.73–42.24)	0.097	1.24 (0.46–3.31)	0.666	1.94 (1.05–3.58)	0.035
**Type of substances**						
Pharmaceutics	1		1		1	
CO/Fumes	10.18 (1.27–21.87)	0.029	2.88 (0.94–8.80)	0.063	3.79 (1.77–8.13)	0.001
Household cleaning products	0.64 (0.34–1.19)	0.158	1.81 (0.96–3.42)	0.067	1.42 (0.98–2.04)	0.060
Cosmetics	0.40 (0.16–1.00)	0.050	0.17 (0.02–1.50)	0.112	0.83 (0.40–1.72)	0.618
Alcohol and recreational drugs	0.98 (0.26–3.67)	0.980	1.09 (0.30–3.91)	0.899	2.06 (1.04–4.09)	0.038
Others ^a^	0.42 (0.22–0.79)	0.008	0.95 (0.42–2.15)	0.907	0.81 (0.51–1.28)	0.37
Intercept	3.317	0.034	0.097	< 0.001	0.266	0.001

## Discussion

Acute intoxication in Pediatrics has been identified as a frequent cause of access to the Emergency Departments worldwide: nevertheless, information on this predictable public health problem remains insufficient. Epidemiological studies for each Region/State might help in defining the situation and to understand how this health issue can be prevented and dealt with [2].

Based on these considerations, we have conducted this retrospective study spanning 6 years. The children acutely intoxicated, equally distributed between males and females, represented the 0.38% of the total number of children accessing the CE-RMH. This value is generally lower than those reported in literature for other Centers out of Italy [9–12], although a recent study from Taiwan described a situation similar to ours [13]. Unfortunately, it is difficult to compare our data to other Italian studies because of the lack of recent reports: no studies have been published on this topic for more than two decades. The low percentage of acute intoxications reported in our study might be biased by the globally elevated number of access to the emergency departments, which may be attributable to differences in organization of the national health services among countries; our study also confirms the Italian habit to go directly to emergency without any previous phone contact with the hospital (about 90%) or the poison center, which might be linked to the fact that access to emergency is free.

Almost all papers related to acute intoxication in childhood reported a bimodal peak of incidence: one around 3 years (linked to the “oral phase” of the psycho-emotional development of child, together with an inapt or neglected storage of the toxic agents) and one around the first part of adolescence [4, 12, 14, 15]. This second peak might be interpreted as a self-harming voluntary poisoning: with the onset of puberty, self-injurious phenomena can start caused by bullying, school problems and love disappointments [8, 16, 17]. Interestingly, our series lacks the second peak in children aged 10–14 (Fig. 1b): we report a 2.3% of suicide attempts, a low percentage if compared with other studies [10, 11, 15, 18–20]. Nevertheless, the small rate of suicides found in our study might be influenced by the upper age limit of the population considered, which reaches up to 14 years.

In our series, voluntary poisoning was 4 times more frequent in girls than in boys, and this trend is in accordance with other studies [9, 20–22]. Namely, a longitudinal epidemiological study of suicide in Italian children reports an average suicide rate over the entire period of observation of 0.91 per 100,000; the global rate was 1.21 for males and 0.59 for females [21] but voluntary intoxication accounted for 4.4% in males and 16.5% in females; the risk of hospitalization significantly increases for cases of intentional exposure: these data confirm that poisoning is one of the most common method of suicide amongst females, while for males the most frequent method was hanging [21].

The most common class of drugs assumed to commit suicide was psychotropics and about 50% of these were benzodiazepines. This data might reflect the easiness by which benzodiazepines are available to children, also because of their diffuse misuse in the general population. The increased use of benzodiazepines in adolescents is a wide spreading social phenomenon also confirmed by the scientific literature [23].

Although in most countries (Taiwan, Iran, Qatar, Turkey, Brazil) the leading cause of intoxications is Pharmaceuticals [13, 15, 18, 24], in our paper we report that three out of five children were intoxicated by Nonpharmaceutical agents, in agreement also with a report led in the Italian North-East 20 years ago [8]. This aspect seems of particular relevance since in these two decades many initiatives have been taken to prevent exposure to household cleansers, such as including labeling and icons on the packaging. The not-decreased ratio might suggest that the intervention has not been sufficient.

The most frequent drugs were painkillers: among the considered categories, they can be bought OTC without medical prescription and are likely the most commonly found in Italian house. In agreement with almost all the other studies regarding pediatric intoxications [2, 9, 10, 15], ingestion was the most common route of exposure. Nevertheless, we registered a noteworthy number (4.8%) of inhalations, principally because of a single mass casualty event of intoxication by carbon monoxide involving 35 children in a public primary school in 2017.

Notably, no hospitalization in the pediatric intensive care unit and no deaths were recorded in our series: to the best of our knowledge only two other papers published in 2019 reported no deaths [2, 13]; many other previous studies worldwide declared at least some cases [4, 7, 12, 15, 18, 19, 24–27].

On top of the limits already described along this discussion, it is necessary to remember that our paper is a retrospective work, and for this reason database might be inaccurate since some variables miss many data; moreover we do not have a global overview of poisoning since many patients stay home and this does not capture non-hospitalised poisoning. In addiction it is difficult to compare the different series because some papers include patients up to 16 or 18 years of age.

Although our data consider acute intoxications in children in our Region, this study is not expressive of Italy as a whole.

## Conclusions

Acute intoxication in childhood still remains a preventable public health problem around the world, including the Italian North-West area; nevertheless, our results would seem to paint a less worrying picture if compared to other countries, mainly when considering the number of acute poisoning as suicide attempts among adolescents, a smaller number of severe intoxications and the absence of deaths. Our study would like to be a stimulus to improve knowledge on this topic, overall in Italy and in Europe where the existing data are very scarce. Last but not least, our results might represent a tool for public health authorities to program specific interventions to further reduce this risk.

## Data Availability

All data are available in Figshare Public repository (Accession number 10.6084/m9.figshare.11382054.v1).
